# Ultrasonographic Diagnosis of Finger Flexor Tendon Hypoplasia in a Child with Phalangeal Agenesis

**DOI:** 10.3390/diagnostics14030257

**Published:** 2024-01-25

**Authors:** Cheng-I Chen, Hong-Yi Lin, Wei-Ting Wu, Ke-Vin Chang, Levent Özçakar

**Affiliations:** 1 Department of Physical Medicine and Rehabilitation, Chung Shan Medical University Hospital, Taichung 40201, Taiwan; cliff0104@hotmail.com (C.-I.C.); peter761217@hotmail.com (H.-Y.L.); 2 Department of Physical Medicine and Rehabilitation, School of Medicine, Chung Shan Medical University, Taichung 40201, Taiwan; 3 Department of Physical Medicine and Rehabilitation, National Taiwan University Hospital, Bei-Hu Branch, Taipei 10845, Taiwan; 4 Department of Physical Medicine and Rehabilitation, College of Medicine, National Taiwan University, Taipei 10048, Taiwan; 5 Center for Regional Anesthesia and Pain Medicine, Wang-Fang Hospital, Taipei Medical University, Taipei 11600, Taiwan; 6 Department of Physical and Rehabilitation Medicine, Hacettepe University Medical School, Ankara 06100, Turkey; lozcakar@yahoo.com

**Keywords:** children, hand, agenesis, ultrasound, sonography

## Abstract

Agenesis and hypoplasia affecting multiple flexor tendons within the same hand represent an exceedingly uncommon occurrence, with no previous studies addressing this condition. This report details a 4-year-old girl with agenesis of the right third and fourth fingers, who sought consultation due to the inability to flex her seemingly unaffected second and fifth fingers. Ultrasound examination revealed substantial thinning of the flexor tendons in the second to fifth digits, with a notable absence of attachment to the middle phalanx. In addition to flexor tendon hypoplasia, hypoplasia of the third and fourth middle phalanges was observed. Hand deformities featuring both finger agenesis and flexor tendon hypoplasia across multiple fingers were exceptionally rare. In such instances, ultrasound, in conjunction with radiography, emerges as the recommended initial imaging tool for comprehensive evaluation of both the phalangeal bones and flexor tendons.

Congenital hand deformities are not rare, with an estimated prevalence of at least 0.23% whereby polydactyly and syndactyly are the most common abnormalities [1]. Furthermore, the absence or hypoplasia of the finger flexor tendon in the fifth digit is a congenital anomaly with a prevalence of about 2.5% based on a cadaveric study [2]. Finger agenesis results from the absence of primordial tissue during embryonic development, leading to the failure of finger formation. This condition affects not only the finger itself, but also the surrounding structures such as tendons and ligaments, eventually contributing to concurrent hand deformities and potential psychosocial impacts. While traditional plain radiography is commonly used for initial bone deformity assessments, concerns about radiation exposure, especially in growing children, are noteworthy. At this point, ultrasonography offers a fast, convenient, and safe alternative for detailed evaluation of soft tissues as well as superficial bones. Despite limitations in scanning deep joints or cartilage and operator dependency, advancements in high-resolution transducers do make them a reasonable tool for assessing hand/wrist deformities. For example, ultrasound has shown promise in assessing the triangular fibrocartilage complex [3] and diagnosing nerve pathologies in the distal hand/wrist [4]. Herein, we present this rare case of a 4-year-old girl with a right-hand deformity since birth for whom the ultrasound evaluation is substantiated.

At 11 months old, she was initially brought to our orthopedic clinic due to a congenital deformity in her right hand. The physical examination revealed symbrachydactyly (short fingers that may be webbed or joined) and clinodactyly (fingers that curve to one side) in the right third and fourth fingers, along with a limited range of motion [1]. Stiffness was observed over the distal (DIP) and proximal (PIP) interphalangeal joints of the second and fifth fingers. The range of motion was preserved over the metacarpophalangeal (MCP) joints and the entire thumb. Radiography revealed hypoplasia of the middle phalanges and curvature in the coronal plane of the distal phalanges over the right third and fourth digits (Figure 1).

At the age of four, the patient presented to our clinic with restricted flexion movement in the right second and fifth fingers despite normal phalangeal bones. Fingers with hypoplastic bones displayed a decreased flexion ability. Ultrasound examination was conducted using a Canon Toshiba Xario 100S machine (Canon Medical Systems USA, Inc., Tustin, CA, USA) equipped with a 14L5 linear array. Figure 2 illustrates the longitudinal palmar view of the second fingers bilaterally. A thin, hyperechoic, fibrillar structure over the hyperechoic bony cortex (indicating the second flexor tendon) was observed. However, it was challenging to distinguish whether it was the flexor digitorum profundus (FDP) or superficialis (FDS). The ossifying epiphysis of the second metacarpal, proximal, and middle phalanges was yet not fused with the diaphysis. The right finger flexor tendon extended from the MCP joint to the PIP joint (Figure 2A). However, it became progressively thinner and more difficult to be traced, as it passed through the PIP joint, suggesting tendon hypoplasia without attachment to the middle phalanx. In contrast, the left flexor tendon was notably thicker (Figure 2B) and identifiable from the MCP joint to the distal middle phalanx area.

Transverse views of distinct segments of the right second finger, from proximal to distal, are shown in Figure 3.

Similar observations were made in the remaining third (Figure 4), fourth (Figure 5), and fifth (Figure 6) finger flexor tendons. Notably, a distinct difference between the right (Figure 5A) and the left (Figure 5B) fourth fingers was observed, with only a small segment of hyperechoic line visualized on the right middle phalanx. This finding indicated a hypoplastic middle phalanx.

Further, while evaluating the patient’s second and fifth fingers on the left side, ultrasound imaging depicted normal tendon appearance over the proximal and middle phalanges. Conversely, the same findings were not observed on the right side, where only a tendon attached to the proximal phalanx was evident. This observation highlighted that the hypoplastic flexor tendon of the right second and fifth fingers did not attach to the middle phalanx, therefore impeding flexion movement. The results not only indicated hypoplasia of the right third and fourth middle phalanges, but also revealed hypoplastic flexor tendons in the right second to fifth fingers. Interestingly, the finger with normal phalanges exhibited a different flexion capacity, compared to the finger with phalangeal agenesis.

In contemporary musculoskeletal practice, ultrasound has become an indispensable tool [5]. When addressing finger-related pathologies, it has proven to be invaluable in detecting common disorders such as mallet finger, sagittal band tear, trigger finger, and ulnar collateral ligament lesions. Moreover, ultrasound serves as a guiding tool to differentiate between acute and chronic conditions and can offer guidance for invasive procedures.

Concerning the volar side of the hand, the FDS and FDP tendons are in strong play. Originating from the anterior compartment of the forearm, both have distinct roles in flexing the PIP and DIP joints, respectively [6]. While the FDP is initially situated deeper than the FDS, after the chiasm, it gradually becomes superficial and inserts into the base of the distal phalanx [2]. In a meta-analysis of 34 studies in 2018, it was reported that the functional absence of the FDS of the fifth finger occurred at a frequency of 7.45%. Additionally, the prevalence of an absent FDS in the fifth finger of cadavers was noted at 2.5% [2]. The congenital defect of the FDP is even more infrequent. In 2020, Belbl et al. [6] identified only eight studies that specifically addressed this rare occurrence. In most instances, the absence of either the FDS or FDP tendons is identified in the fifth finger; however, there were cases of missing FDPs in fingers other than in the fifth digit [6,7]. For the detection of finger tendon absence or hypogenesis, ultrasound facilitates to trace tendon continuity and/or potential impingement.

Symbrachydactyly, which originally refers to an anomaly with short fingers and abnormal linkages between adjacent digits, is now more often associated with a spectrum of sporadic and unilateral hand malformations [1]. Clinodactyly is characterized by an angulation of the finger in the radioulnar plane, with the fifth digit being typically affected [1]. The typical embryonic development of the hand initiates as the extremity buds flatten between days 34 and 38. The separation of digits commences around day 50, through a sequence of signal modulations. Subsequently, the tendon and pulley systems undergo complete maturation between days 60 and 80 [8].

Due to the absence of flexor tendons, our patient experienced difficulty in performing finger flexions at the PIP and DIP joint levels. Herein, ultrasound has proven beneficial in distinguishing the cause, i.e., being related to bone, joint, pulley, or flexor tendon problems [9]. Additionally, there are essential considerations when conducting ultrasound examination for pediatric finger abnormalities. First, attention should be given to the growth plate pattern and the ossification center of the epiphysis. Second, it is noteworthy that girls may exhibit an advanced bone age of 1–2 years relative to boys of the same chronological age [10]. In the hand, fusion of epiphysis to metaphysis takes place between 12 and 18 years in girls and between 16 and 18 years in boys. This process follows a distinct pattern, with fusion initiating in the distal phalanges, followed by metacarpals, proximal phalanges, and finally middle phalanges. All hand bones reach complete development after the onset of puberty [10]. A scoring system was introduced to assess ultrasonography images, where grades 1 to 5 correspond to the following conditions respectively: smaller epiphysis compared to diaphysis, epiphysis equal to diaphysis, sesamoid visibility, capping, and fusion [11]. Needless to say, it is crucial not to misinterpret the distinctive developmental features of pediatric patients as pathological changes, e.g., fractures.

In conclusion, high-frequency probes have revolutionized ultrasound, making it an invaluable tool for swiftly and comprehensively evaluating both normal anatomy and diverse finger pathologies. This imaging modality provides significant insights as regards flexor tendons, bony cortex, and joints. Particularly for prompt examination of rare/multiple hand deformities with finger agenesis and flexor tendon hypoplasia, ultrasound would be the recommended imaging tool in addition to conventional radiography.

## Figures and Tables

**Figure 1:**
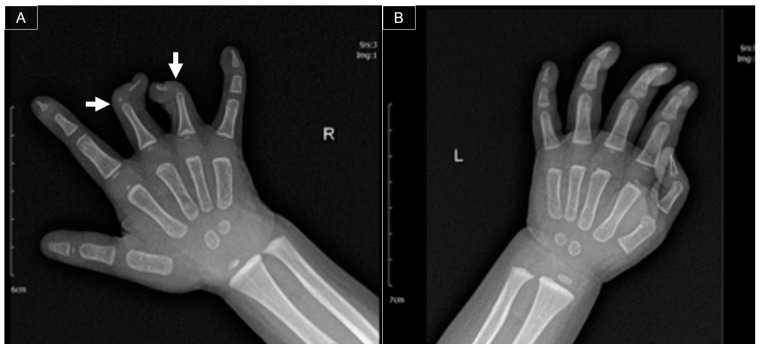
Bilateral hand X-rays ((**A**) right hand; (**B**) left hand) indicating hypoplasia in the middle phalanges and angulation deformities (arrows) in the distal phalanges of the right third and fourth fingers.

**Figure 2:**
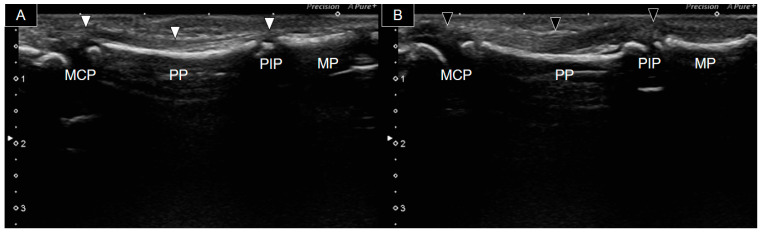
Ultrasound image of the palmar side of the second right (**A**) and left (**B**) fingers. The right flexor tendon (white arrowheads) was significantly thinner than the left one (black arrowheads) and was not visible over the right middle phalanx. MCP, metacarpophalangeal joint; PP, proximal phalanx; PIP, proximal phalangeal joint; MP, middle phalanx.

**Figure 3:**
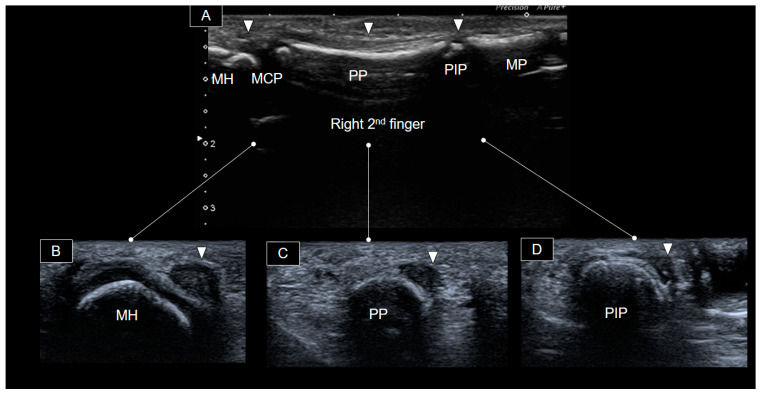
Ultrasound images depicting the palmar aspect of the right 2nd finger in the long axis (**A**) and at distinct segments (arrowheads) progressing from proximal to distal in the short axis (**B**–**D**). MCP, metacarpophalangeal joint; PP, proximal phalanx; PIP, proximal phalangeal joint; MP, middle phalanx; MH, metacarpal head.

**Figure 4:**
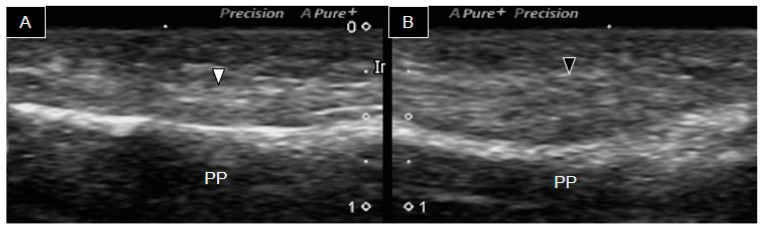
Ultrasound image of the palmar side of the third right (**A**) and left (**B**) fingers. The white arrowhead indicates the right finger flexor tendon, whereas the black arrowhead indicates the left flexor tendon. PP, proximal phalanx.

**Figure 5:**
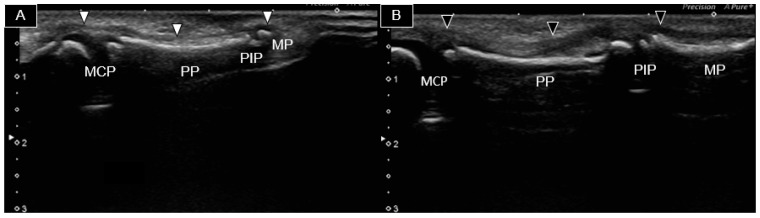
Ultrasound image of the palmar side of the fourth right (**A**) and left (**B**) fingers. The white arrowhead indicates the right finger flexor tendon, whereas the black arrowhead indicates the left flexor tendon. MCP, metacarpophalangeal joint; PP, proximal phalanx; PIP, proximal phalangeal joint; MP, middle phalanx.

**Figure 6:**
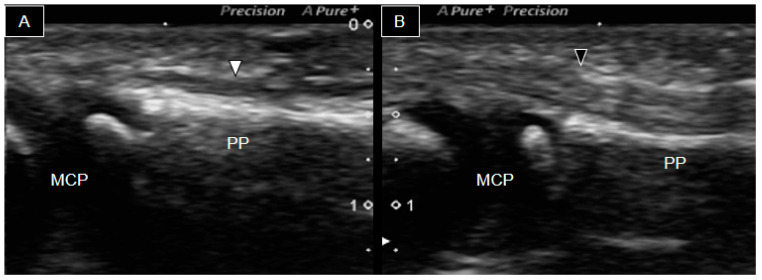
Ultrasound image of the palmar side of the fifth right (**A**) and left (**B**) fingers. The white arrowhead indicates the right finger flexor tendon, whereas the black arrowhead indicates the left flexor tendon. MCP, metacarpophalangeal joint; PP, proximal phalanx.

## Data Availability

Data are contained within the main text of the manuscript.
